# Update on comparative genome mapping between *Malus *and *Pyrus*

**DOI:** 10.1186/1756-0500-2-182

**Published:** 2009-09-14

**Authors:** Jean-Marc Celton, David Chagné, Stuart D Tustin, Shingo Terakami, Chikako Nishitani, Toshiya Yamamoto, Susan E Gardiner

**Affiliations:** 1 University of Western Cape, Biotechnology Department, Modderdam Road, Bellville, Cape Town, 7535, South Africa; 2 The New Zealand Institute for Plant & Food Research Limited, Palmerston North 4442, New Zealand; 3 The New Zealand Institute for Plant & Food Research Limited, Hawke's Bay, PB 1401, Havelock North 4157, New Zealand; 4 National Institute of Fruit Tree Science, Tsukuba, Ibaraki 305-8605, Japan

## Abstract

**Background:**

Comparative genome mapping determines the linkage between homologous genes of related taxa. It has already been used in plants to characterize agronomically important genes in lesser studied species, using information from better studied species. In the Maloideae sub-family, which includes fruit species such as apple, pear, loquat and quince, genome co-linearity has been suggested between the genera *Malus *and *Pyrus*; however map comparisons are incomplete to date.

**Findings:**

Genetic maps for the apple rootstocks 'Malling 9' ('M.9') (*Malus *× *domestica*) and 'Robusta 5' ('R5') (*Malus *× *robusta*), and pear cultivars 'Bartlett' and 'La France' (*Pyrus communis*) were constructed using Simple Sequence Repeat (SSR) markers developed from both species, including a new set of 73 pear Expressed Sequence Tag (EST) SSR markers. Integrated genetic maps for apple and pear were then constructed using 87 and 131 SSR markers in common, respectively.

The genetic maps were aligned using 102 markers in common, including 64 pear SSR markers and 38 apple SSR markers. Of these 102 markers, 90 anchor markers showed complete co-linearity between the two genomes.

**Conclusion:**

Our alignment of the genetic maps of two *Malus *cultivars of differing species origin with two *Pyrus communis *cultivars confirms the ready transferability of SSR markers from one genus to the other and supports a high level of co-linearity within the sub-family Maloideae between the genomes of *Malus *and *Pyrus*.

## Findings

Comparative genomics involves the assessment of the degree of genomic conservation between species to enable the transfer of genetic information, such as the position of major genes, quantitative trait loci (QTL), and candidate genes, in order to validate their role. Practically, comparative genome mapping determines the linkage between homologous genes of related taxa by aligning genome maps using orthologous molecular markers, which can greatly assist in genetic analysis of less-studied species. It is often measured by the degree of synteny, that is, the conservation of gene content, and co-linearity, which represents the conservation of gene order between conserved genomic regions. The first example of genome comparison in plants was made between tomato and potato and used conserved restricted fragment length polymorphism (RFLP) probes [1] to reveal a high degree of co-linearity between these species.

The Maloideae sub-family includes fruit species such as apple, pear, loquat and quince. Apple (*Malus *× *domestica*) and pear (*Pyrus *species) are major temperate fruit crops that have been cultivated in Europe and Asia for at least 2,000 to 3,000 years [2]. Molecular markers linked to a number of agronomically important traits, such as resistance to pests and diseases, tree architecture, and fruit quality, have been identified in both apple [3] and pear [4]. Their identical chromosome number (2n = 2x = 34) and similar genome size (apple 1.57 pg/2C [5]; pear 1.11 pg/2C) [6], as well as their supposed recent divergence date (33.9 to 55.8 million years ago [7]) suggests that their genomes might be highly co-linear. When co-linearity between *Malus *and *Pyrus *was examined by comparing maps of the European pears (*Pyrus communis*) 'Bartlett' and 'La France', and the Japanese pear (*Pyrus pyrifolia*) 'Housui' with those of apple cultivars (*Malus *× *domestica*) 'Fiesta' and 'Discovery' [8] using 66 transferable apple simple sequence repeat (SSR) loci, all the pear linkage groups were aligned to the apple consensus linkage groups with at least one marker in common [9]. These results showed that positions of SSR loci are well conserved between apple and pear, suggesting that partial co-linearity exists between the genera. A study by [10] reported that more than 100 apple SSR markers could be positioned on pear genetic linkage maps. [11] described the mapping of six pear SSR loci on the linkage maps of apples 'Fiesta' and 'Discovery', and their location in homologous linkage groups. This was confirmed in a later study based on a larger set of pear SSR markers mapped in a 'Malling 9' × 'Robusta 5' ('M.9' × 'R5') apple rootstock population [12].

Here we report on a detailed comparison of the apple and pear genomes using a new set of transferable SSR markers developed from pear coding sequences.

## Methods

### Plant material

The genetic maps that were used as framework maps for positioning the new markers have previously been described. Construction of the genetic map of the European pear cultivars 'La France' and 'Bartlett' is described in [9]. The apple rootstock genetic linkage maps constructed in 'M.9' (*Malus *× *domestica*) and 'R5' (*Malus *× *robusta*) are described in [12]. The new SSR markers were screened over 60 F_1 _individuals from the 'M.9' × 'R5' population, consisting of the bin mapping set [12] of 14 plants together with 46 additional seedlings.

### SSR markers

Seventy-three new SSR markers developed from pear EST were tested for their amplification and segregation in the 'M.9' × 'R5' apple population. These markers are prefixed by TsuENH and their PCR primer sequence and accession of the EST are given in Table 1. PCR amplifications were performed as described in [13] with the modifications described in [12]. PCR fragments were separated as described in [9] and [12] for pear and apple, respectively.

**Table 1:** List of pear EST-based SSR markers tested in apple.

SSR locus	accession	Location on pear consensus map	Location on apple consensus map	Forward primer (5'-3')	Reverse primer (5'-3')
TsuENH001	AB450689	LG2	**LG2**	AAAGACGGCATTGACTGGATAGA	gtttcttGATGCAAAGACTTTCGCCTATCT

TsuENH004	AB450692	LG4, 12	**LG4**	CGCATTAAAGTCTGGCTTTCTTC	gtttcttGAATTGGCAGAGAGATTGAGTGG

TsuENH008	AB450696	LG9	**LG13**	CTGAGGTCTCATTCGGTGATTCT	gtttcttCCTTCTCTGCTTTCTTCTTCACG

TsuENH011	AB450698	LG6, 14	**LG6**	CGCTTATCCGTTAAACTTCA	gtttcttCAACGACAGTTCGAATAGGA

TsuENH016	AB450701	LG15	**LG15**	TCATTTCATGGACTCTCAATCTCC	gtttcttCGAGGAGTCTGTCTGCGTCT

TsuENH022	AB450705	LG16	**LG16**	CCAATTCATCGAAGTTTACATAGGG	gtttcttGGCCAAAGTGCATGAGATGTAGT

TsuENH023	AB450706	LG3	**LG3**	TCTTCTTCACAGGTCACAACAGC	gtttcttCTATGCGCTGCTTCTTGGTAGTC

TsuENH031	AB450711	LG14	**LG14**	CATTTCCTTAGCTCCCTCCAGTT	gtttcttGAACCTTCCTCTTCCCATCACTT

TsuENH032	AB450712	LG14	**LG14**	AACTGGAGGGAGCTAAGGAAATG	gtttcttCATCAAACAAAACTAGCCGAACC

TsuENH033	AB450713	LG17	**LG17**	CCTGAGGTTATTGACCCAAAAGA	gtttcttGGTGGATACTCACTCAGTTGGAAA

TsuENH034	AB450714	LG8	**LG8**	GCCCCCAATATTTTCCCATTAT	gtttcttGTTGGTGTTTGAGTCAACGTGAG

TsuENH042	AB450718	LG13, 16	**LG16**	AAAGCCGTACATTAGGCAAACC	gtttcttAAAACTAGAACAGGCAGCCACAG

TsuENH043	AB450719	LG10	**LG10**	CATCTGCGTCCGGCAAAC	gtttcttGCCCCCTGATATTCGTGGAT

TsuENH046	AB450722	LG6	**LG6**	GGTCATCACCCACTTAAAAACCA	gtttcttGTGCCCTGAAGTAATTGAGATGG

TsuENH049	AB450724	LG1	**LG1**	CATCAGCCTACGAGCACATACAC	gtttcttAGATTACGGCAACAGCAACAGAT

TsuENH052	AB450726		**LG13, LG16**	CCCAGCAGCCTCCTAATCAATA	gtttcttTAGTTGTAGTCCTGCCCAAGTCC

TsuENH058	AB450730	LG14	**LG14**	AGAAGAAGGATAAGAAGAAGGATGG	gtttcttGTAACGAAAAGGAAACAGGACTTG

TsuENH060	AB450732		**LG10**	ATCTCCACCCAAGAAACCTTACC	gtttcttGGGGGTGAGGAATAGATGTAACC

TsuENH062	AB450733	LG2	**LG2**	ACTCAGATCGTACGCAGAACAAA	gtttcttCGATAAAGATCGATAATCCTCATGC

TsuENH066	AB450736		**LG13**	GTGGTGGAGGTGCGTATTGAC	gtttcttCGAGGAAAAGTGCGACTCGT

TsuENH068	AB450738		**LG5**	CCAATTTTCTCTTCCTCCCTGTT	gtttcttTTACAGTTATTGCCGAAGCCAAG

TsuENH076	AB450743		**LG10**	CATTAATACGCTGCTGTTTCTGC	gtttcttACTTGAATTGGGGTAGGGATTGT

TsuENH079	AB450745	LG16	**LG13**	GCGGCTTCTTGGGAGAAGGT	gtttcttGCATGCTCCTTTTGACAGCCTAC

TsuENH081	AB450747		**LG13**	GCTCTCCTCTTCTTCTCCCACTC	gtttcttCCACCCTCGTCAAAATCAGAGTA

TsuENH082	AB450748		**LG11**	CACCAGTACTCCTGGAGGGTTTC	gtttcttGTGCTCCTGCAACATTTTCTCC

TsuENH083	AB450749	LG11	**LG11**	ACTCTCCGCAAAACAATGTCGTA	gtttcttTGTGAGAGTTTGAGGAGGAGAGC

TsuENH086	AB450751	LG5	**LG5**	CTCTGTTCTGCTTCGATTCTGCT	gtttcttGTCCACGTTCACCATTTTTCAGT

TsuENH093	AB450757	LG15	**LG8, LG15**	GTGGAGATTTTCCGAGTCAAATG	gtttcttAATAAGACTGCTGAGGGAATCCA

TsuENH096	AB450759	LG15	**LG8**	AGTGAGAGAGAGAGGCCTTGGTT	gtttcttGCTCTTGCTCTGTCTTCGAAATG

EST: expressed sequence tag. SSR: single sequence repeat. LG: linkage group.

### Linkage analysis and map integration

The new EST-based SSR markers were positioned on the parental apple and pear genetic maps using JoinMap v3.0 software [14] with a minimum LOD score of 5.0 for grouping using the Kosambi function. Integrated consensus genetic maps were constructed by merging the 'M.9' and 'R5' datasets for apple, and the 'La France' and 'Bartlett' datasets for pear, using the "combine" function of JoinMap v3.0. This merging method is based on the calculation of mean recombination frequencies and combined LOD scores. Graphical representations of the maps were drawn using MapChart v2.0 [15]. For the integration of the female and male maps, SSR markers in common among homologous linkage groups were used for the analysis, and all apple SSR markers mapped on pear, as well as all pear SSR markers mapped on apple, were included. Other types of markers, such as amplified fragment length polymorphisms (AFLP), single nucleotide polymorphisms (SNP), sequence characterized amplified regions (SCAR), and isozymes were removed from the map, except when located in large gaps between SSR markers, or at the extreme end of a linkage group.

## Results

### Pear EST-SSR polymorphism

All 73 pear SSR markers developed from pear expressed sequence tags (EST) amplified a PCR product when tested on apple and 29 (40%) were polymorphic in the 'M.9' × 'R5' population (Table 1). Two markers, TsuENH052 and TsuENH093, amplified two loci in apple. Nineteen and 22 pear EST-SSR markers were mapped on 'M.9' and 'R5', respectively, bringing the total number of pear markers mapped on 'M.9' to 51 and 'R5' to 61 [12]. The integrated consensus 'M.9' × 'R5' map contained a total of 96 SSR loci derived from 90 primer pairs developed from either pear genomic DNA or EST.

### Genetic map integration and comparison

Integrated consensus maps for apple ('M.9' and 'R5') and pear ('La France' and 'Bartlett') were constructed using 87 and 131 markers in common between the respective parents of the mapping populations. The 'M.9' × 'R5' population generated a map of 1,230 cM, whereas the integrated map of pear was shorter, at 1,146 cM.

**Figure 1:**
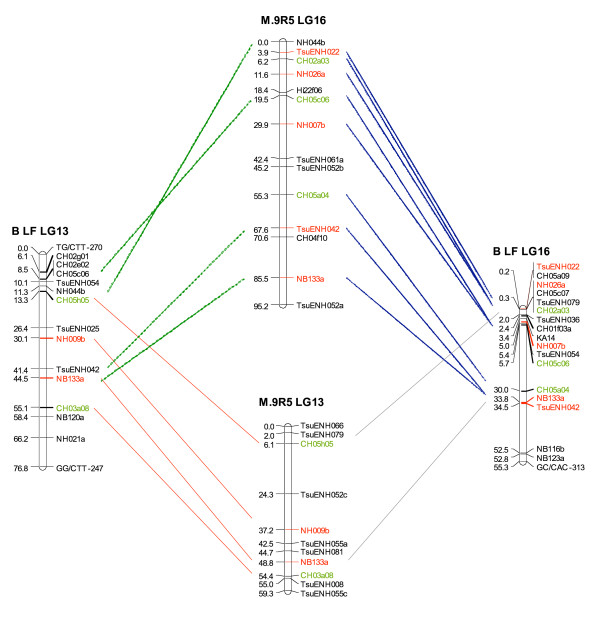
**Alignment of pear and apple linkage groups (LG) 13 and 16 showing inter-specific co-linearity and intra-specific LG homeology**. Alignment of the apple (M.9 R5) and pear (B LF) consensus genetic maps. SSR markers in common between the linkage groups are linked to each other with a line and are presented in color. SSR markers developed from apple sequences are in green while SSR markers developed from pear sequences are in red. M.9: 'Malling 9'. R5: 'Robusta 5'; B: 'Bartlett'. LF: 'La France'. SSR: single sequence repeat.

The alignment of the integrated pear and apple maps was performed using 102 SSR markers in common (Additional file 1). Of these 102 markers, 90 (53 pear and 37 apple SSR) mapped at similar locations on homologous linkage groups in apple and pear, and three mapped in what are considered to be homoeologous linkage groups (i.e. LG 13 and LG 16; Figure 1). Eight SSR markers in common between apple and pear were mapped in non-homologous and non-homoeologous linkage groups (Table 2).

**Table 2:** SSR markers mapping in non-homologous and unknown homoeologous linkage groups in apple and pear

	Apple LG	Pear LG
CH-Vf1	1	10

NH041a	5/10	7

NH027a	6	15

NB104a	9	12

NH045	11	10

TsuENH008	13	9

NB111a	15	11

NH013a	17	1

SR: single sequence repeat. LG: linkage group.

## Discussion

### Apple and pear genome synteny

Alignment of the genetic linkage maps made it possible to validate a high degree of co-linearity between the apple and pear genomes (Additional file 1). All pear linkage groups could be successfully aligned to the 'M.9' × 'R5' consensus map by at least one SSR marker. The whole of the pear LG 8 and LG 15 could be aligned to their apple homologues using five and six SSR markers, respectively. Other pear linkage groups, such as LG 2, LG 4, LG 10, LG 14 and LG 16 were partially aligned to the apple consensus map (Additional file 1). Other portions of the genomes, such as LG 7, the top of LG 3 and LG 6, and the bottom of LG 12 and LG 14 still remain incompletely aligned, and these should become the focus of future research aimed at aligning the complete apple and pear genomes at the level of genetic markers.

In most instances, the order and distances between individual markers were similar for apple and pear, suggesting the presence of regions that are highly conserved between the two genomes. Some SSR markers demonstrated multi-locus amplification across the two genera, such as CH05c06, NH044b, NB133a, TsuENH042, TsuENH079 and TsuENH052, that mapped on both LG 13 and LG 16 in both apple and pear (Figure 1). A few apparent inversions in marker order can be observed in Additional file 1: (i) LG 1, KA4b is above TsuENH049 in pear; (ii) LG 2, TsuENH062 is above BGT23b in pear; (iii) LG 9, CH05c07 and CH01f03b are inverted in pear with respect to apple; (iv) LG 11, CH04h02 and TsuENH083 are inverted in pear; (v) LG 12, NH207a is above NZ28f04 in pear. These apparent inversions may be a consequence of the relatively small number of individuals used to map these markers, 55 to 63 individuals in the pear population, and 60 individuals for apple. The number of progeny individuals genotyped in the mapping populations will need to be increased in future studies in order to validate these possible marker inversions. Overall, a high level of co-linearity between apple and pear genetic maps was confirmed.

As shown in Table 2, eight SSR markers mapped to non-homologous and possible uncharacterized homoeologous linkage groups in apple and pear. Fragments obtained from PCR amplification of four loci (NH013a, NH045a, NB104a and TsuENH008) using apple DNA were cloned and sequenced (data not shown). Their sequences were checked for the presence of a SSR motif and then compared to the original pear sequence when available. The amplification products obtained in apple using NH013a, NH045a and TsuENH008 contained a SSR motif, whereas NB104a did not. The sequence for pear and apple NH013a and NH045a were highly similar, except at the level of the SSR motif, which was shorter for apple than pear for both loci. The apparent change in map location does not correspond to unspecific PCR amplification, but could have resulted from small regions of genome re-organization in apple and pear. However, the current density of markers in common between the two integrated maps presented in this study does not allow such an interpretation of the results for the present.

### Genome synteny in the Maloideae

Extensive comparative mapping studies in plant families such as Solanaceae, Poaceae and Brassicaceae have consistently indicated that genome evolution within a family consists mainly of limited chromosome rearrangements, leading to the conservation of large chromosome fragments. Similar results have already been found in the Rosaceae within the genus *Prunus *[16,17], and have resulted in the complete alignment of the peach and apricot genomes [18]. Within the Maloideae, the results of our study confirm the high level of co-linearity between *Malus *and *Pyrus *genomes proposed by [2]. It is possible that further comparative mapping studies among other members of the Maloideae, such as loquat and quince, will demonstrate that all members of this family should be considered as a single genetic system. SSR markers from *Malus *have been used to construct the first genetic linkage map of loquat (*Eriobotrya japonica*) [19] and to conduct a diversity study in quince (*Cydonia oblonga*) [20].

These findings could be the precursor to the development of a set of genetic markers common to all members of the Maloideae. Markers with multi-locus amplification across two genera, such as observed for CH05c06, NH044b, NB133a, TsuENH042, TsuENH079 and TsuENH052 that mapped on homeologous linkage groups LG 13 and LG 16 in both apple and pear (Figure 1), would be invaluable for this purpose. A comprehensive marker set would enable a more efficient identification of loci for disease resistance and quality traits using comparative genome mapping across the Maloideae.

## Conclusion

We have extended the list of SSR markers used for comparative genome mapping in the Maloideae, specifically for *Malus *and *Pyrus*. As the apple whole genome sequence will be available soon, we propose that apple should be used as the model for species in the Maloideae and that genetic information can be inferred in the other species by comparison of their genetic maps with that of apple. Furthermore, we suggest that this approach may be extended to include other members of the Rosaceae, such as *Prunus*, *Rubus *and *Rosa*.

## Competing interests

The authors declare that they have no competing interests.

## Authors' contributions

JMC tested and screened the pear SSR in apple; performed the genetic mapping analysis and map comparison, designed these experiments and prepared the manuscript. DC contributed to the experiments and the preparation of the manuscript. SDT provided plant material for the apple ('M.9' × 'R5') mapping population. ST, CN and TY developed the pear SSR markers and the reference maps for pear (B LF). SEG was project leader. All authors have read and approved the manuscript.

## Supplementary Material

Additional file 1 **Alignment of the 17 linkage groups (LG) of apple and pear**. The figure provided represents the alignment of the apple (M.9 R5) and pear (B LF) consensus genetic maps. SSR markers in common between the maps are linked to each other with a black line and are presented in color. SSR markers developed from apple sequences are in green while SSR markers developed from pear sequences are in red. M.9: 'Malling 9'. R5: 'Robusta 5'; B: 'Bartlett'. LF: 'La France'. SSR: single sequence repeat. Click here for file
