# Supplementary figures and images for: Update on comparative genome mapping between Malus and Pyrus
Source: BMC Res Notes. 2009 Sep 14;2:182. doi: 10.1186/1756-0500-2-182 (PMC2749866; doi:10.1186/1756-0500-2-182)

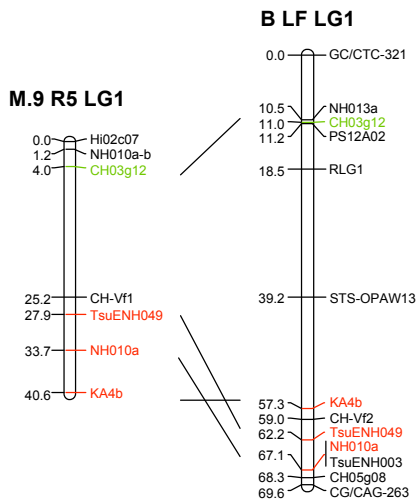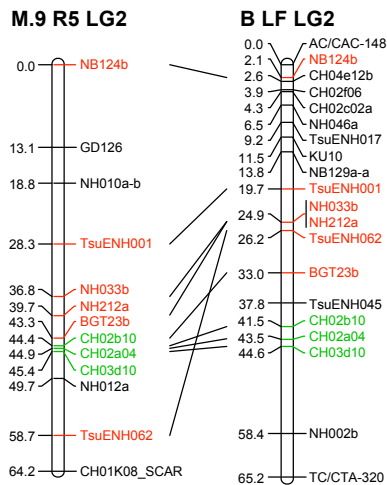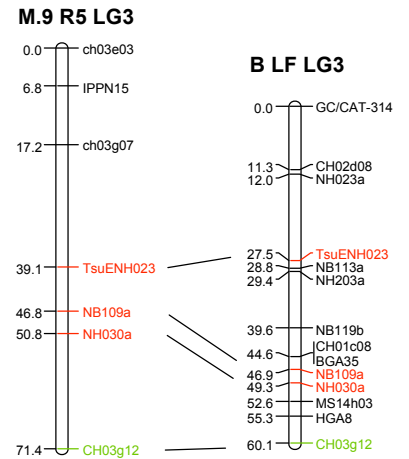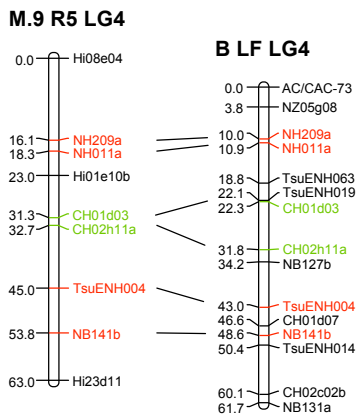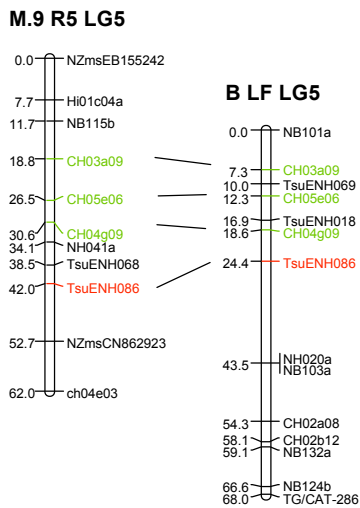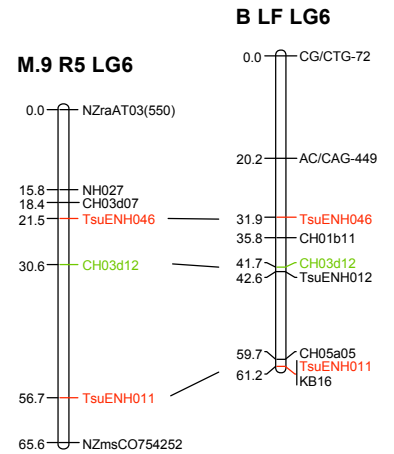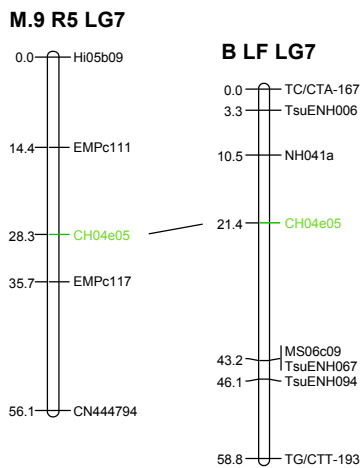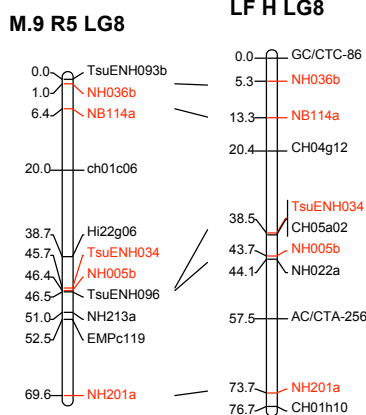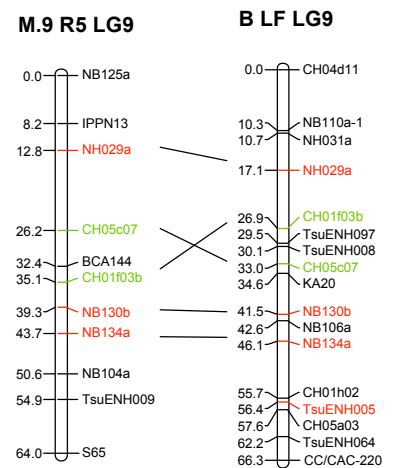

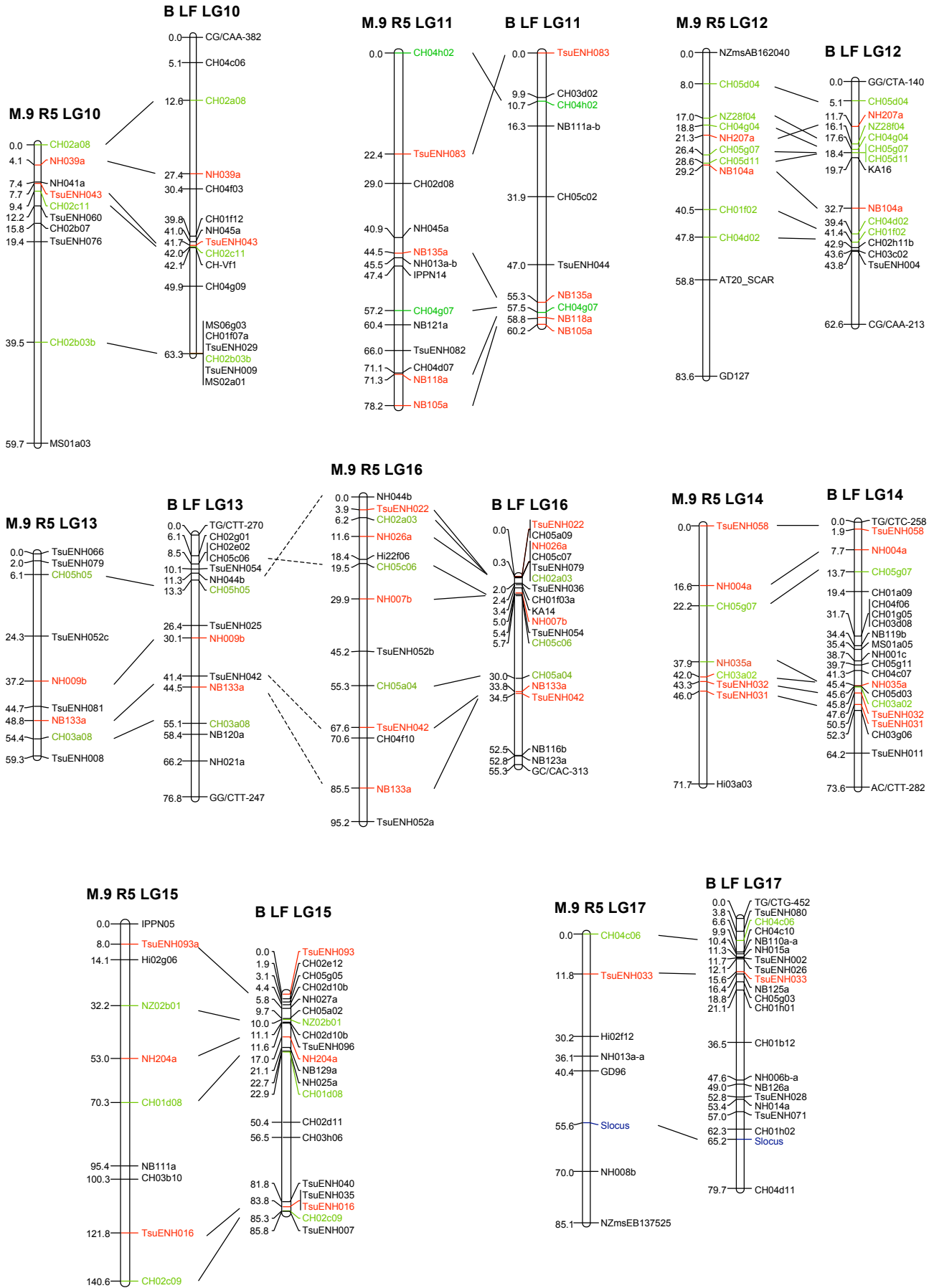

Supplement: Additional file 1 — Alignment of the 17 linkage groups (LG) of apple and pear. The figure provided represents the alignment of the apple (M.9 R5) and pear (B LF) consensus genetic maps. SSR markers in common between the maps are linked to each other with a black line and are presented in color. SSR markers developed from apple sequences are in green while SSR markers developed from pear sequences are in red. M.9: 'Malling 9'. R5: 'Robusta 5'; B: 'Bartlett'. LF: 'La France'. SSR: single sequence repeat. [file 1756-0500-2-182-S1.pdf]
